# PKNOX2 suppresses gastric cancer through the transcriptional activation of IGFBP5 and p53

**DOI:** 10.1038/s41388-019-0743-4

**Published:** 2019-02-11

**Authors:** Li Zhang, Weilin Li, Lei Cao, Jiaying Xu, Yun Qian, Huarong Chen, Yanquan Zhang, Wei Kang, Hongyan Gou, Chi Chun Wong, Jun Yu

**Affiliations:** 1 Institute of Digestive Disease and Department of Medicine and Therapeutics, State Key Laboratory of Digestive Disease, Li Ka Shing Institute of Health Sciences, CUHK-Shenzhen Research Institute, The Chinese University of Hong Kong, Hong Kong, Hong Kong; 2 Department of Surgery, The Chinese University of Hong Kong, Hong Kong, Hong Kong; 3 Department of Gastroenterology, Shenzhen University Hospital, Shenzhen, China; 4 Department of Anatomical and Cellular Pathology, The Chinese University of Hong Kong, Hong Kong, Hong Kong

**Keywords:** Gastric cancer, Cancer genetics

## Abstract

Promoter methylation plays a vital role in tumorigenesis through transcriptional silencing of tumor suppressive genes. Using genome-wide methylation array, we first identified PBX/Knotted Homeobox 2 (PKNOX2) as a candidate tumor suppressor in gastric cancer. PKNOX2 mRNA expression is largely silenced in gastric cancer cell lines and primary gastric cancer via promoter methylation. Promoter methylation of PKNOX2 was associated with poor survival in gastric cancer patients. A series of in vitro and in vivo functional studies revealed that PKNOX2 functions as a tumor suppressor. Ectopic PKNOX2 expression inhibited cell proliferation in GC cell lines and suppressed growth of tumor xenografts in mice via induction of apoptosis and cell cycle arrest; and suppressed cell migration and invasion by blocking epithelial-to-mesenchymal transition. On the other hand, knockdown PKNOX2 in normal gastric epithelial cells triggered diverse malignant phenotypes. Mechanistically, PKNOX2 exerts its tumor suppressive effect by promoting the up-regulation of Insulin like Growth Factor Binding Protein 5 (IGFBP5) and TP53. PKNOX2 binds to the promoter regions of IGFBP5 and TP53 and transcriptionally activated their expression by chromatin immunoprecipitation (ChIP)-PCR assay. IGFBP5 knockdown partly abrogated tumor suppressive effect of PKNOX2, indicating that the function(s) of PKNOX2 are dependent on IGFBP5. IGFBP5 promoted PKNOX2-mediated up-regulation of p53. As a consequence, p53 transcription target genes were coordinately up-regulated in PKNOX2-expressing GC cells, leading to tumor suppression. In summary, our results identified PKNOX2 as a tumor suppressor in gastric cancer by activation of IGFBP5 and p53 signaling pathways. PKNOX2 promoter hypermethylation might be a biomarker for the poor survival of gastric cancer patients.

## Introduction

Gastric cancer (GC) is the fifth most common cancer worldwide and the third leading cause of cancer-related mortality with 723,000 deaths per year [1]. GC is asymptomatic in the early stages, and about 80–90% of GC patients are diagnosed at an advanced stage [2]. As a consequence, the overall five-year survival rate is low (~20%). Thus, it remains important to identify functional biomarkers for diagnosis and prognosification of GC.

DNA methylation is an important epigenetic mechanism in the development of GC. Numerous tumor suppressor genes have been shown to be repressed by hypermethylation in cancers [3–6]. DNA methylation silences tumor suppressor gene expression by directly interfering with binding of transcription factors to specific site(s) in the promoter region; or indirectly by recruiting methyl-CpG binding domain proteins. Epigenetic silencing of gene expression through promoter hypermethylation is a useful epigenetic marker for identification of novel tumor suppressor genes. Using Illumina 450 K DNA methylation array, we identified PBX/Knotted Homeobox 2 (PKNOX2) as a novel gene differentially methylated in GC.

PKNOX2 belongs to the Three Amino acid Loop Extension (TALE) class of homeodomain proteins characterized by a 3-amino acid extension between alpha helices 1 and 2 within the homeodomain. The TALE family consists of PBX (PBX1-4), MEIS (MEIS1-3), and PKNOX (PKNOX1-2). The TALE family of proteins is sequence-specific transcription factors that share a conserved DNA-binding domain and they play fundamental roles in growth, differentiation and death; and have also been implicated in tumorigenesis [7–10]. PKNOX2 is located on the chromosome 11q24.2. Previous studies demonstrated the wide spread expression of PKNOX2 during organogenesis and in the adult, which suggests that PKNOX2 participates in diverse developmental processes [11]. PKNOX2 has also been found to be expressed in melanoma, but was silenced in human tumor cell lines from various tissues [12]. However, the expression, biological role and the clinical significance of PKNOX2 in GC remain elusive.

Here, we conducted the first study on PKNOX2 in GC. We identified frequent silencing of PKNOX2 via promoter methylation in GC cell lines and primary GC tissues. We revealed that PKNOX2 possesses tumor suppressive effects in GC cells and inhibits GC growth by inducing cell apoptosis and cell cycle arrest, and inhibiting metastasis in vitro and in vivo. Tumor suppressive effect of PKNOX2 is mediated by transcriptional activation of IGFBP5 and p53 tumor suppressive pathways. Finally, we found that PKNOX2 promoter methylation predicts poor outcomes in GC patients.

## Results

### 450 K methylation array identified PKNOX2 promoter hypermethylation in human GC

**Fig. 1:**
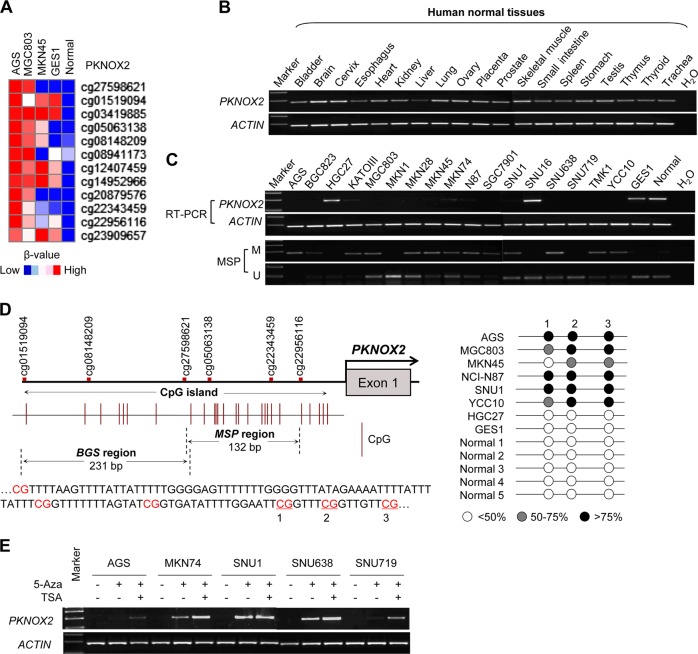
PKNOX2 expression and promoter methylation in GC cell lines. **a** Infinium HumanMethylation450BeadChip revealed that PKNOX2 was preferentially methylated in GC cell lines. **b** PKNOX2 mRNA levels in human normal tissues, as determined by RT-PCR. **c** PKNOX2 mRNA expression (*upper*) and promoter methylation (*lower*) in GC cells. Methylation specific PCR (MSP) was performed to detect PKNOX2 methylation (M: methylated; U: unmethylated). **d** CpG island on the PKNOX2 promoter. The regions for bisulfite sequencing (BGS) and MSP are shown. Each vertical bar represents a single CpG. TSS: transcription start site. PKNOX2 is methylated in GC cell lines compared to normal gastric cell line and tissues. **e** PKNOX2 mRNA expression was restored upon demethylation treatment with 5-Aza

We profiled the methylome of three GC cell lines (AGS, MGC803, and MKN45), one normal gastric cell line (GES1), and one normal gastric tissue using the Infinium Human Methylation450BeadChip (450 K) assay. As shown in Fig. 1a, we revealed that PKNOX2 was preferentially methylated in GC. PKNOX2 was hypermethylated in all three GC cell lines (AGS, MGC803 and MKN45) as compared to GES1 cells and normal gastric tissues.

### PKNOX2 is silenced in GC cell lines via promoter methylation

We first determined PKNOX2 mRNA expression in 19 human normal tissues by RT-PCR. As shown in Fig. 1b, PKNOX2 mRNA was expressed in most human normal tissues including normal stomach. Next, we assessed PKNOX2 mRNA expression in 17 GC cell lines, GES1 and one normal gastric tissue by RT-PCR (Fig. 1c). PKNOX2 mRNA was silenced in 15 out of 17 GC cell lines; while it was readily expressed in GES1 cells and normal gastric tissues, suggesting that PKNOX2 is specifically silenced in GC. We next investigated the promoter methylation status in GC cell lines by methylation-specific PCR (MSP). PKNOX2 methylation can be detected in GC cell lines with silencing of PKNOX2, whereas PKNOX2 promoter was largely unmethylated in cell lines expressing PKNOX2 (HGC27, SNU16, and GES1) and normal gastric tissues (Fig. 1c). We also performed bisulfite sequencing (BGS) (Fig. 1d) which demonstrated PKNOX2 promoter methylation (>75%) in GC cell lines. On the other hand, HGC27 (expressing PKNOX2), GES1 and normal gastric tissues were largely unmethylated. To verify the relationship between PKNOX2 mRNA expression and promoter methylation, 5 randomly selected PKNOX2-silenced GC cell lines (AGS, MKN74, SNU1, SNU638, and SNU719) were treated with 5-Aza (an inhibitor of DNA methyltransferase) with or without TSA (an inhibitor of histone deacetylase) (Fig. 1e). PKNOX2 mRNA expression was restored by 5-Aza treatment, indicating that promoter hypermethylation mediates the transcriptional silencing of PKNOX2 in GC cells. Collectively, our data suggest that PKNOX2 was down-regulated in GC cell lines through aberrant promoter methylation.

### PKNOX2 is silenced by promoter hypermethylation in primary GC

**Fig. 2:**
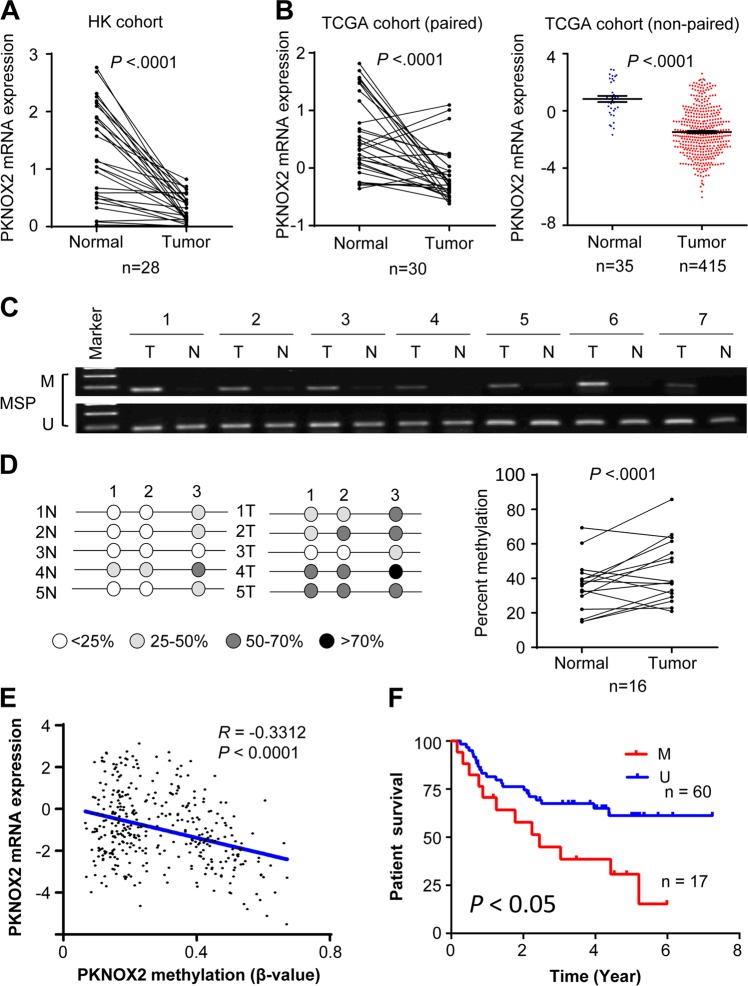
PKNOX2 expression and promoter methylation in primary GC. **a** PKNOX2 mRNA expression was down-regulated in 28 pairs of primary GC compared with adjacent normal tissues in the Hong Kong GC cohort (*P* < 0.0001). **b** PKNOX2 mRNA expression in paired and non-paired GC samples from TCGA database (*P* < 0.0001). **c** MSP and (**d**) BGS analysis demonstrated PKNOX2 promoter methylation in primary GC compared with adjacent normal tissues (*P* < 0.0001). **e** PKNOX2 promoter methylation was inversely associated with its mRNA expression in TCGA GC cohort (*R* = -0.3312, *P* < 0.0001). **f** PKNOX2 promoter methylation was correlated with poor survival in GC patients (*P* < 0.05)

To evaluate the clinical significance of PKNOX2 in human GC, we examined mRNA expression of PKNOX2 in 28 pairs of GC tissues and adjacent normal gastric tissues from Hong Kong cohort by real-time-PCR. PKNOX2 mRNA was down-regulated in primary GC tissues compared with adjacent normal tissues (*P* *<* 0.0001) (Fig. 2a). We also analyzed mRNA expression of PKNOX2 in both paired tissues (*n* = 30) and non-paired GC tissues from TCGA database. Consistently, PKNOX2 mRNA expression was decreased in GC (*P* *<* 0.0001) compared with normal gastric tissues (Fig. 2b). We next determined the promoter methylation of PKNOX2 in primary GC with MSP and BGS. MSP revealed the ubiquity of promoter methylation in GC tissues (Fig. 2c), whilst adjacent normal tissues were unmethylated. BGS confirmed that PKNOX2 promoter methylation in tumors were significantly higher than that of adjacent normal tissues (*P* < 0.0001) (Fig. 2d). Moreover, analysis of TCGA cohort revealed a negative association (*R* = −0.3312) between PKNOX2 mRNA and promoter methylation (Fig. 2e). These results indicated that PKNOX2 was silenced in primary GC and its down-regulation was associated with promoter hypermethylation.

### PKNOX2 hypermethylation is associated with poor outcomes in GC

In order to assess the prognostic value of PKNOX2 in GC, we analyzed the correlation between PKNOX2 methylation and patient survival in the Beijing GC cohort. With the best cut-off value determined by ROC curve analysis, patients with high PKNOX2 methylation in GC tumors had significantly shorter survival than those with low PKNOX2 methylation (*P* < 0.05) (Fig. 2f). In the TCGA cohort, PKNOX2 hypermethylation was also predicted with poor patient survival in GS subtype GC (Supplementary Fig. 2). These results suggest that PKNOX2 hypermethylation predicts poor prognosis in GC patients.

### PKNOX2 suppresses GC cell growth

**Fig. 3:**
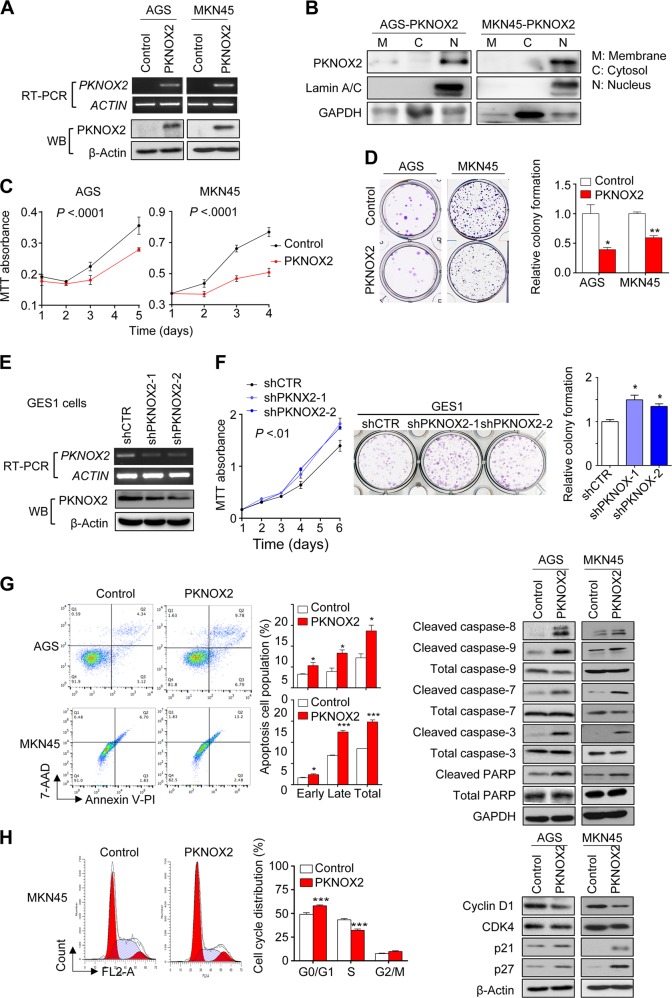
PKNOX2 suppressed GC cell proliferation via induction of apoptosis and cell cycle arrest. **a** PKNOX2 overexpression in AGS and MKN45 cells was confirmed by RT-PCR and Western blot. **b** Subcellular localization of PKNOX2 in AGS and MKN45 cells. **c** PKNOX2 overexpression suppressed cell proliferation (both *P* < 0.0001) and (**d**) colony formation(AGS, *P* < 0.05;MKN45,*P* < 0.001). **e** Knockdown of PKNOX2 in GES1 cells was determined by RT-PCR and Western blot. **f** PKNOX2 silencing promoted GES1 cell growth (*P* < 0.01)and colony formation (*P* < 0.05). **g** PKNOX2 induced apoptosis in AGS and MKN45 cells. Apoptosis was determined by flow cytometry after Annexin V-PI/7-AAD staining (*left*)(AGS, *P* = 0.0179;MKN45, *P* = 0.0002). Apoptosis markers activated by PKNOX2 were determined by Western blot (*right*). **h** PKNOX2 increased cell population in G_0_/G_1_ phase(*P* < 0.0001), together with decreased S phase population (*left*)(*P* < 0.0001). PKNOX2 inhibited the expression of cyclin D1 and CDK4; but induced p21 and p27 expression (*right*). (*P < 0.05; **P < 0.001; ***P < 0.0001)

To explore biological function of PKNOX2 in GC, we transfected PKNOX2 expression vector or empty vector into AGS and MKN45 cells. Overexpression of PKNOX2 was confirmed at the mRNA and protein levels using RT-PCR and Western blot, respectively (Fig. 3a). Consistent with its functional role as a transcription factor, PKNOX2 was mainly localized to the nucleus (Fig. 3b).

We then performed MTT and colony formation assays to assess the effect of PKNOX2 on GC cell growth. PKNOX2 overexpression in AGS and MKN45 cells significantly decreased cell viability (both *P* *<* 0.0001; Fig. 3c). Ectopic expression of PKNOX2 also significantly inhibited colony formation efficiency (AGS, *P* *<* 0.05; MKN45, *P* *<* 0.001; Fig. 3d). Knockdown of PKNOX2 in the normal gastric cell line GES1 with two independent shRNA (Fig. 3e) led to increased cell proliferation (*P* *<* 0.01) and colony formation (*P* *<* 0.05) (Fig. 3f). Hence, PKNOX2 inhibits GC cell proliferation, suggesting that it functions as a tumor suppressor.

### PKNOX2 promotes apoptosis and blocks cell cycle progression

To determine the mechanisms by which PKNOX2 suppressed GC cell growth, we performed apoptosis assay by flow cytometry. As shown in Fig. 3g, both early and late apoptotic cell populations were significantly increased in AGS and MKN45 cells expressing PKNOX2 (AGS, *P* = 0.0179; MKN45, *P* = 0.0002) compared with controls. Next, we detected the expression of several apoptosis markers and found that PKNOX2 induced cleavage of caspase-8, caspase-9, caspase-7, caspase-3, and poly(ADP-ribose) polymerase (PARP), suggesting that PKNOX2 promoted apoptosis in GC cells.

We then tested the effect of PKNOX2 on cell cycle progression in GC cell lines (Fig. 3h). In MKN45 cells, ectopic expression of PKNOX2 slowed cell cycle progression by inducing G_0_/G_1_ arrest, thereby decreasing their transition to the S phase. Consistent with this, we found that PKNOX2 down-regulated protein levels of G_1_ cell cycle promoters cyclin D1 and CDK4, while simultaneously enhancing the expression of G_1_ cell cycle inhibitors p21 and p27.

### PKNOX2 suppresses GC cell migration and invasion

**Fig. 4:**
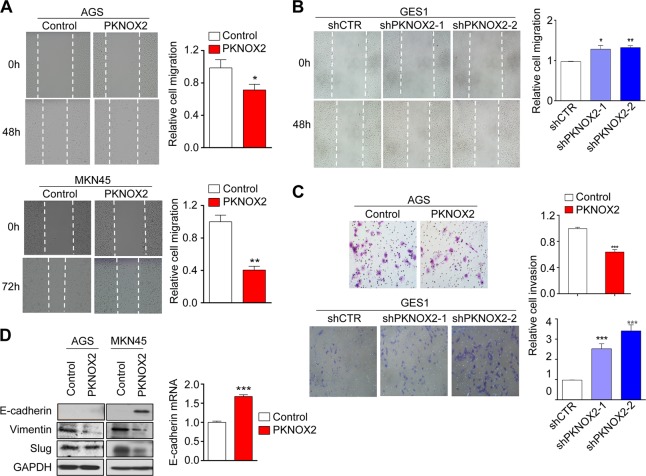
PKNOX2 suppressed gastric cancer cell migration/invasion in vitro. **a** PKNOX2 overexpression suppressed GC wound closure in AGS and MKN45 cells (AGS, *P* < 0.05; MKN45, *P* < 0.01), whilst (**b**) PKNOX2 knockdown in GES1 cells had an opposite effect (shPKNOX2-1, *P* = 0.0141; shPKNOX2-2, *P* < 0.01). **c** PKNOX2 overexpression in AGS cells suppressed cell invasion(*P* < 0.001); while silencing of PKNOX2 in GES1 cells promoted cell invasion(both *P* < 0.01). **d** PKNOX2 negatively regulated EMT, indicated by increased expression of E-cadherin and decreased expression of Vimentin and Slug. *P < 0.05; **P < 0.001; ***P < 0.0001)

We performed the wound healing assay to determine the effect of PKNOX2 on GC cell invasion. A delay in wound closure was observed in AGS (*P* *<* 0.05) and MKN45 (*P* *<* 0.01) cells overexpressing PKNOX2 (Fig. 4a); whereas the silencing of PKNOX2 in GES1 promoted wound closure (shPKNOX2-1, *P* = 0.0141; shPKNOX2-2, *P* *<* 0.01) (Fig. 4b). Matrigel invasion assay was next performed to assess the effect of PKNOX2 on GC cell invasion. Ectopic expression of PKNOX2 in AGS cells significantly decreased the number of cells that migrated through the invasion chamber (*P* *<* 0.001; Fig. 4c). Conversely, the number of invading cells were increased after knockdown of PKNOX2 in GES1 cells (both *P* *<* 0.01; Fig. 4c). These results indicate that PKNOX2 suppressed GC cell migration and invasion.

We next asked whether the effect of PKNOX2 on cell migration/invasion was a consequence of the epithelial-to-mesenchymal transition (EMT). EMT markers such as E-cadherin,Vimentin and slug were examined by Western blot. As shown in Fig. 4d, PKNOX2 induced the expression of E-cadherin, while downregulating Vimentin and Slug expression in AGS and MKN45 cells. While we could not detect E-cadherin protein in AGS cells, its mRNA expression was increased (Fig. 4d). This indicates that PKNOX2 suppressed EMT in GC.

### Ectopic expression of PKNOX2 depresses tumor growth in vivo

**Fig. 5:**
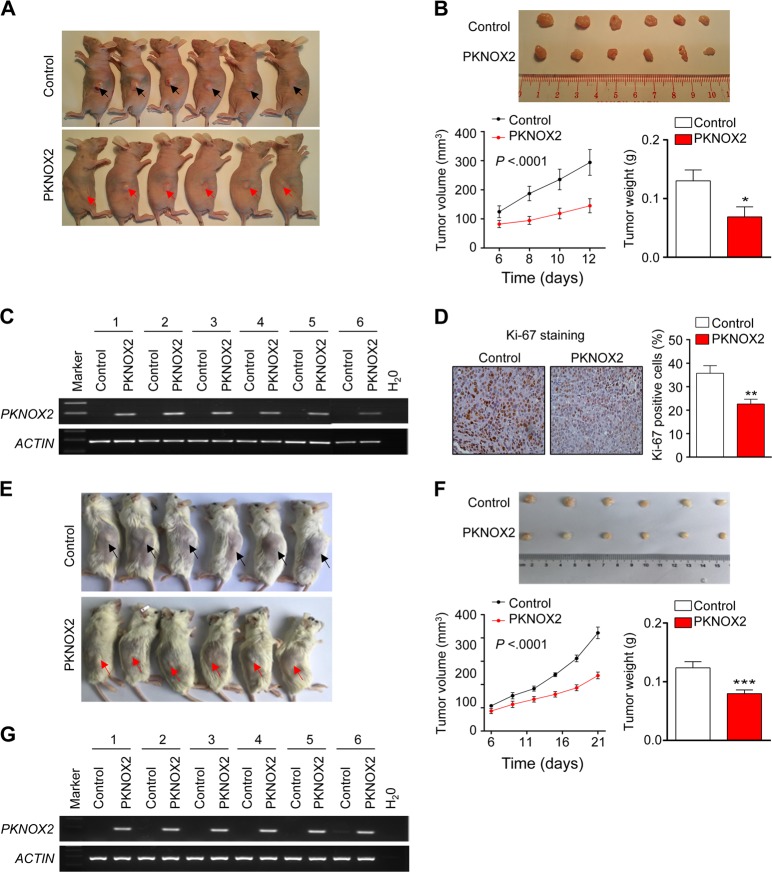
PKNOX2 suppressed gastric tumor growth in vivo. **a** Ectopic expression of PKNOX2 suppressed the growth of MKN45 xeongrafts in vivo. **b** Representative image of MKN45 xenografts in nude mice (*upper*). PKNOX2 overexpression reduced both tumor volume (*P* < 0.001) and weight (*P* < 0.05) (*lower*). **c** Overexpression of PKNOX2 in MKN45 xenografts was confirmed by RT-PCR. **d** PKNOX2 inhibited cell proliferation in vivo, as evidenced by Ki-67 staining(*P* < 0.01). **e** Ectopic expression of PKNOX2 suppressed the growth of AGS xeongrafts in NOD-SCID mice. **f** Representative image of AGS xenografts (upper). PKNOX2 overexpression reduced both tumor volume and weight (lower)(both *P* < 0.001). **g** Overexpression of PKNOX2 in AGS xenografts was confirmed by RT-PCR. (*P < 0.05; **P *<* 0.01; ***P < 0.0001)

To further confirm the biological function of PKNOX2 in GC, we performed in vivo tumorigenicity assay. MKN45 cells stably transfected with PKNOX2 vector or empty vector were repectively injected into right and left flanks of nude mice (Fig. 5a). Tumor size was significantly smaller in PKNOX2 overexpression group than that in control group (*P* *<* 0.001, Fig. 5b). Besides, the average tumor weight in PKNOX2 overexpression group was significantly reduced compared to control group (*P* *<* 0.05, Fig. 5b). This data demonstrated that PKNOX2 functions as a tumor suppressor in GC in vivo. Ectopic expression of PKNOX2 in tumor tissues from nude mice was confirmed by RT-PCR (Fig. 5c). Cell proliferation in the tumor tissues from nude mice was evaluated by Ki-67 immunostaining assay, and the PKNOX2 group displayed decreased cell proliferation compared to control group (*P* *<* 0.01, Fig. 5d). We next examined the effect of PKNOX2 overexpression on AGS cell growth in vivo. NOD-SCID mice were injected with AGS cells expressing empty vector or PKNOX2. As shown in Fig. 5e, f, PKNOX2 overexpression significantly suppresed tumor growth and reduced tumor weight at the end point (both *P* < 0.001). PKNOX2 overexpression was validated by RT-PCR (Fig. 5g). Collectively, these findings indicate that PKNOX2 suppresses tumor growth in GC.

### IGFBP5 is a transcriptional target of PKNOX2

**Fig. 6:**
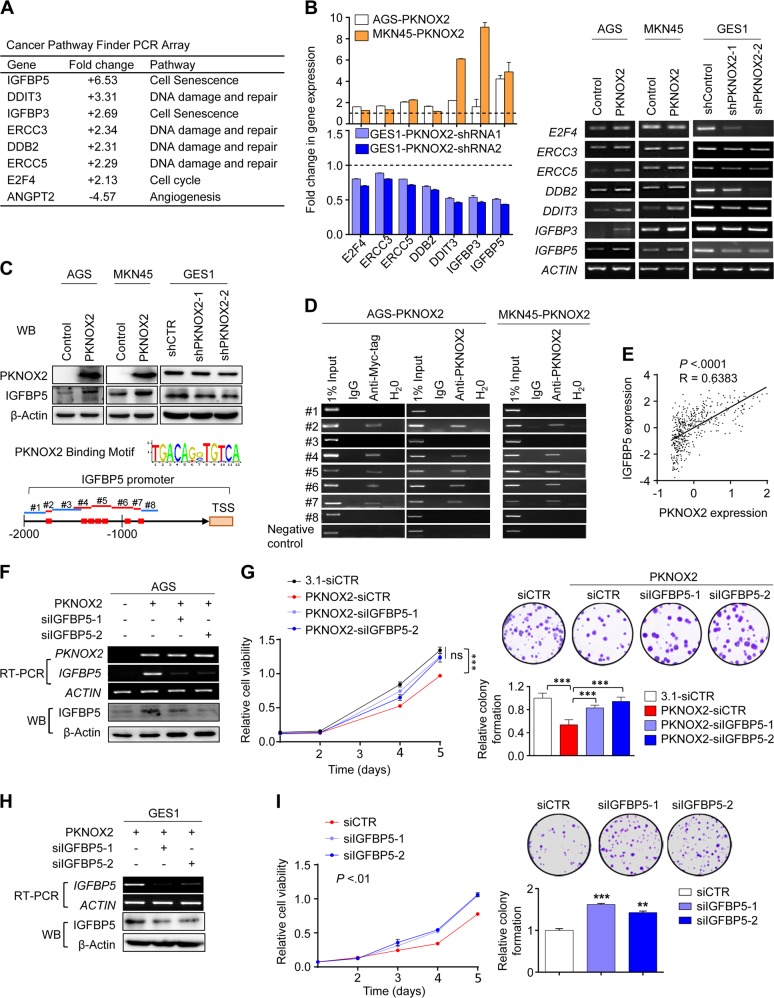
IGFBP5 is a transcriptional target of PKNOX2 in GC. **a** PKNOX2 regulated genes were identified by Human Cancer PathwayFinder PCR Array. Genes with <0.5-fold or >2-fold change in AGS overexpressing PKNOX2 as compared with control AGS cells. **b** PCR validation of differentially expressed genes by real-time PCR and RT-PCR, respectively. **c** PKNOX2 positively regulated IGFBP5 protein expression. **d** PKNOX2 directly binds to IGFBP5 promoter. Binding motif for PKNOX2 was predicted by JASPAR database (red squares). ChIP-PCR using anti-Myc-tag or anti-PKNOX2 revealed direct binding of PKNOX2 to the promoter region of IGFBP5. **e** Correlation between the expression of PKNOX2 and IGFBP5 in GC patients from the TCGA cohort (*P* < 0.001, *R* = 0.6383). **f** IGFBP5 knockdown in AGS cells overexpressing PKNOX2 was validated by RT-PCR and Western blot. **g** The tumor suppressive effect of PKNOX2 was partly dependent on IGFBP5. IGFBP5 knockdown partly abolished the growth inhibitory effect of PKNOX2 on cell viability and colony formation in AGS cells (both *P* < 0.0001). **h** Knockdown of IGFBP5 in GES1 cells. **i** IGFBP5 silencing in GES1 cells promoted cell viability (*P* < 0.01) and colony formation in GES1 cells(siIGFBP5-1, *P* < 0.0001;siIGFBP5-2, *P* < 0.01). (**P *<* 0.01; ***P *<* 0.0001)

Cancer Pathway Finder PCR array was performed in AGS cells overexpressing PKNOX2 and control vector, which revealed a number of significantly altered genes (<0.5-fold or >2-fold) (Fig. 6a). Among these genes, the insulin-like growth factor binding protein 5 (IGFBP5) was the top outlier gene with >6.5-fold up-regulation in PKNOX2-overexpressing AGS cells. Apart from IGFBP5, several genes (DDIT3, ERCC3, ERCC5, and DDB2) involved in DNA damage and repair were concomitantly up-regulated. Up-regulation of these genes by PKNOX2 overexpression was validated by real-time PCR and RT-PCR in AGS and MKN45 cells (Fig. 6b). Up-regulation of IGFBP5 upon PKNOX2 overexpression was also confirmed at the protein level by Western blot in AGS and MKN45 cells (Fig. 6c). Conversely, PKNOX2 knockdown in GES1 cells down-regulated IGFBP5 at both mRNA and protein levels (Fig. 6b, c). Based on these findings, we hypothesized that tumor suppressive effect of PKNOX2 in GC might be mediated by IGFBP5.

Given that PKNOX2 functions as a transcription factor, we hypothesized that PKNOX2 might directly promote IGFBP5 transcription. We first performed in silico prediction based on the binding motif for PKNOX2 in JASPAR database (Fig. 6d). ChIP-PCR assay [13] using anti-Myc-Tag and anti-PKNOX2 was then performed to assess the direct interaction of PKNOX2 with IGFBP5 promoter (Fig. 6d). Based on in silico prediction, we designed eight pair of primers containing potential PKNOX2 binding sites. Positive bands were observed in putative binding regions (2 and 4); while no bands were found in adjacent nonbinding regions (Fig. 6d) in AGS-PKNOX2 and MKN45-PKNOX2 cells. No bands were observed in anti-PKNOX2 pulldown in AGS and MKN45 empty vector cells (Supplementary Fig. 3). Moreover, ChIP-seq confirmed the interaction of PKNOX2 in close proximity to IGFBP5 promoter (Supplementary Fig. 4). These data indicated that IGFBP5 is a transcriptional target of PKNXO2 in GC. To further evaluate the relationship between PKNXO2 and IGFBP5, we analyzed the association between PKNOX2 and IGFBP5 mRNA expression in TCGA GC cohort (Fig. 6e). Indeed, PKNOX2 expression positively correlated with IGFBP5 (*P* *<* 0.001, *R* = 0.6383, Fig. 6e). Collectively, PKNOX2 positively regulates IGFBP5 expression in vitro and in vivo.

### Tumor suppressive function of PKNOX2 is dependent on IGFBP5

IGFBP5 is a putative tumor suppressor [14, 15]. We thus investigated whether IGFBP5 functions downstream of PKNOX2. We silenced IGFBP5 in AGS cells with ectopic expression of PKNOX2 using two independent siRNAs. IGFBP5-siRNAs reversed PKNOX2-mediated IGFBP5 mRNA and protein expression (Fig. 6f). Moreover, cell viability and colony formation assays revealed that IGFBP5 silencing abrogated the tumor suppressive effect of PKNOX2 in AGS cells overexpressing PKNOX2 (Fig. 6g), suggesting that tumor suppressive function of PKNOX2 in GC cells was dependent on IGFBP5. We next assess if IGFBP5 has a tumor suppressive role in GES1 cells that express endogenous PKNOX2. IGFBP5 knockdown in GES1 cells (Fig. 6h) significantly induced cell viability (*P* < 0.01) and colony formation (*P* < 0.01) (Fig. 6i). IGFBP5 thus operates as a tumor suppressor in GC cells expressing PKNOX2.

### PKNOX2 activates p53 signaling pathway

**Fig. 7:**
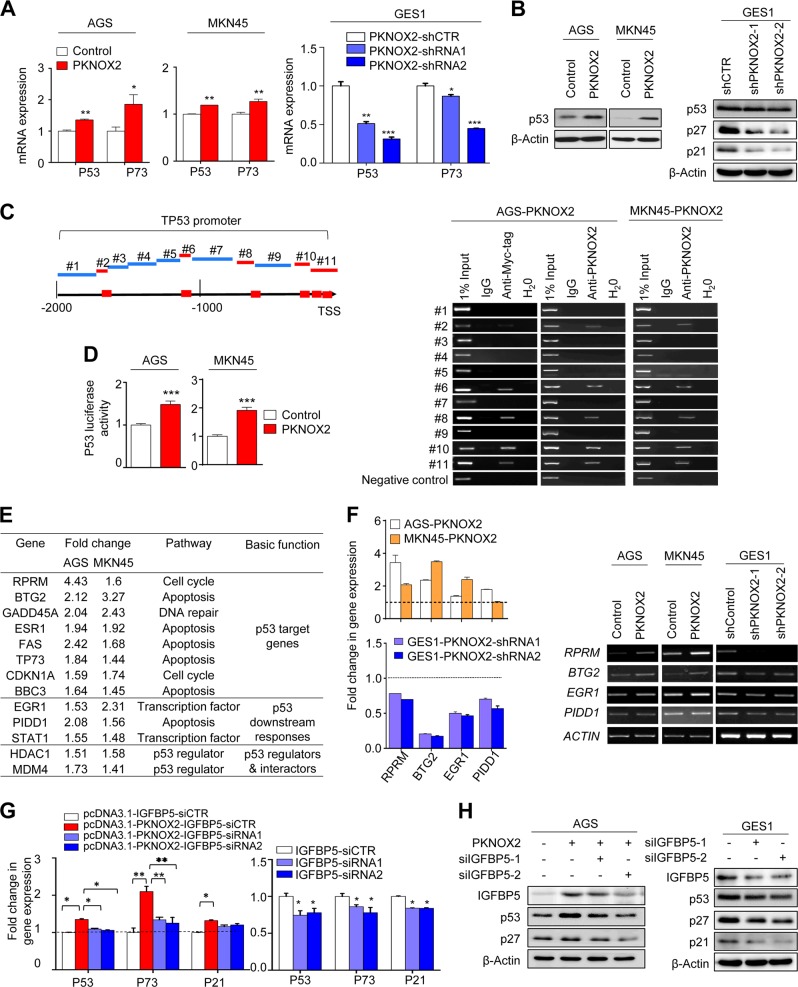
IGFBP5 activates p53 signaling pathway in GC. **a** p53 mRNA and (**b**) protein was up-regulated by PKNOX2 overexpression in AGS and MKN45 cells; while an opposite trend was observed for GES1 cells. **c** PKNOX2 binds to the promoter of TP53. Binding motif of PKNOX2 was predicted by JASPAR database (red squares). ChIP-PCR validated the interaction between PKNOX2 and p53 promoter region. **d** PKNOX2 induced p53 luciferase activity in AGS and MKN45 cells(both *P* < 0.0001). **e** p53 signaling pathway PCR array showed that PKNOX2 overexpression up-regulated numerous p53 target genes in AGS and MKN45 cells. **f** Validation of differentially expressed genes by real-time PCR and RT-PCR. **g** and **h** Knockdown of IGFBP5 partly abolished the induction of p53 by PKNOX2 in AGS cells, as evidenced by real-time PCR and Western blot. IGFBP5 knockdown also down-regulated p53, p21 and p27 expression in GES1 cells. (*P *<* 0.05; **P *<* 0.01; ***P *<* 0.0001)

Given that a number of p53-targeted genes associated with DNA repair were positively regulated by PKNOX2, we next investigated whether PKNOX2 can affect p53 signaling pathway. Both p53 and p73 were up-regulated at mRNA levels by overexpression of PKNOX2 in AGS and MKN45 cells (Fig. 7a); and p53 protein was also up-regulated by PKNOX2 (Fig. 7b). Consistently, knockdown of PKNOX2 down-regulated protein expression of p53, p21, and p27 in GES1 cells (Fig. 7a, b). In silico analysis revealed putative binding sites for PKNOX2 on the p53 promoter (Fig. 7c). ChIP-PCR using anti-Myc-tag or anti-PKNOX2 revealed the presence of multiple PKNOX2 binding sites in the p53 promoter (Fig. 7c). No bands were observed in anti-PKNOX2 pulldown in AGS and MKN45 empty vector cells (Supplementary Fig. 3). In addition, ectopic expression of PKNOX2 significantly activated p53 luciferase reporter activities in AGS and MKN45 cells (Fig. 7d). These results indicate that PKNOX2 directly regulates p53 activity.

To confirm activation of p53, we performed p53 signaling pathway PCR array. In both AGS and MKN45 cells, overexpression of PKNOX2 was associated with up-regulation of a panel of p53 target genes and several genes involved in p53 downstream responses (Fig. 7e), which was further confirmed by real-time PCR and RT-PCR analyses (Fig. 7f). The up-regulated genes were associated with apoptosis induction (BTG2, TP73 and PIDD1) and cell cycle arrest (RPRM and CDNK1A), consistent with the tumor suppressive function of PKNOX2.

### Activation of p53 by PKNOX2 is partly dependent on IGFBP5

Previous research indicated that IGFBP5 exerts a tumor suppressive effect via the activation of p53. We thus investigated whether IGFBP5 might contribute to the activation of p53 by PKNOX2. Indeed, knockdown of IGFBP5 in AGS cells ectopically expressing PKNOX2 partly abolished induction of p53 (Fig. 7g, h). PKNOX2-mediated expression of p53 downstream factors, such as p21 and p27, were partly reversed upon IGFBP5 knockdown (Fig. 7g, h). In GES1 cells expressing endogenous PKNOX2, knockdown of IGFBP5 also inhibited expression of p53, p21, and p27 (Fig. 7g, h). Our findings thereby indicated that IGFBP5 represents an additional mechanism through which PKNOX2 activates the p53 tumor suppressive signaling pathway.

## Discussion

As a member of TALE family of proteins, PKNOX2 functions as a transcription factor. However, its role in cancer remains unknown. Here, we demonstrated that PKNOX2 is readily expressed in diverse normal tissues, but is aberrantly silenced in GC via promoter methylation at high frequency. PKNOX2 promoter hypermethylation is associated with poor outcomes in GC patients, suggesting that PKNOX2 could function as a tumor suppressor in GC.

Consistent with our hypothesis, the ectopic expression of PKNOX2 in GC cells (AGS and MKN45) suppressed cell proliferation and colony formation; while its silencing in normal gastric GES1 cells, which express endogenous PKNOX2, had opposite effects. PKNOX2 exerted tumor suppressive effects by inhibiting cell proliferation, migration and invasion. PKNOX2 promoted apoptosis via the engagement of the intrinsic (mitochondrial) apoptosis pathway, as evidenced by sequential activation caspase-9, caspase-7, caspase-3, and PARP. PKNOX2 also triggered G_1_ cell cycle arrest, which was associated with the inhibition of cyclin D1/CDK4 and the induction of p21/p27. Cyclin D1/CDK4 is a protein complex integral to G_1_/S progression, whereas p21/p27 are cell cycle inhibitors that mediate CDK inhibition [16]. Moreover, PKNOX2 suppressed cell migration/invasion via EMT blockade. E-cadherin, critical for maintaining cell adhesion and cellular polarity [17, 18], was up-regulated by PKNOX2; while markers of cell motility/invasion Slug and Vimentin was simultaneously down-regulated [19, 20]. Collectively, these results indicate that PKNOX2 functions as a tumor suppressor in GC.

To elucidate the mechanisms of action of PKNOX2, we performed expression profiling, which led to identification of IGFBP5 as a target of PKNOX2. IGFBP5 expression was profoundly up-regulated (>6-fold) by PKNOX2 in GC cell lines. ChIP-PCR analysis confirmed direct binding of PKNOX2 to IGFBP5 promoter. In addition, PKNOX2 expression was positively correlated to that of IGFBP5 in TCGA GC cohort. Hence, in vitro and in vivo evidence indicate that IGFBP5 is a transcriptional target of PKNOX2 in GC. Recent studies have demonstrated a tumor suppressor role of IGFBP5. IGFBP5 induced apoptosis and cell cycle arrest in breast cancer cells [14]; and it inhibited the tumorigenicity of head and neck squamous cell carcinoma in vivo and in vitro [21]. IGFBP5 was also a bio-marker for predicting patient prognosis and response to chemotherapy [22–25]. Consistent with the above observations, our data demonstrated that the tumor suppressive effect of PKNOX2 depends on IGFBP5, as knockdown of IGFBP5 partly abolished the tumor suppressive effect of PKNOX2 in GC cells.

Our expression profiling also showed that PKNOX2 activated a p53-dependent DNA damage response. PKNOX2 induced the expression and transcriptional activity of p53, a classical tumor suppressor. ChIP-PCR showed that PKNOX2 can interact with p53 promoter to mediate its expression. In concordance with this discovery, p53 transcriptional target genes were coordinately up-regulated upon ectopic PKNOX2 expression; whilst PKNOX2 knockdown reduced their expression. These p53 target genes are involved in cell cycle, apoptosis and DNA repair pathways. Notable targets up-regulated by PKNOX2 include BTG2, a tumor suppressor with anti-proliferative and anti-metastatic properties [26, 27], and RPRM, which has been reported to suppress tumorigenesis in gastric cancer [28]. Our data revealed significant cross-talk between IGFBP5 and p53, as knockdown of IGFBP5 partly abrogated PKNOX2-induced p53 expression, suggesting that IGFBP5 might play a role in reinforcing p53 activation in GC cells expressing PKNOX2. Consistent with our observations, Kim et al. found that IGFPB5 induced cellular senescence in HUVEC cells via a p53-dependent mechanism [29]. IGFBP5 might also induce the activity of p53 by promoting its phosphorylation, thereby contributing to p53 stabilization [30]. Taken together, IGFBP5 and p53 might act in concert to mediate the tumor suppressive effect of PKNOX2 in GC.

**Fig. 8:**
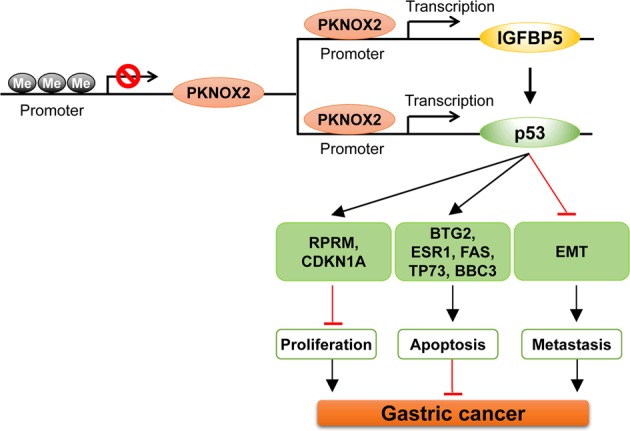
The schematic illustration of the molecular mechanism of PKNOX2 in GC

In summary, our study indicated for the first time that PKNOX2 functions as a candidate tumor suppressor in GC by induction of apoptosis, inhibition of cell cycle progression and blockade of EMT, culminating in inhibition of cell growth, migration/invasion in vitro and tumorigenicity in vivo. Tumor suppressive effect of PKNOX2 is mediated via IGFBP5 and activation of p53 signaling pathway, with IGFPB5 reinforcing p53 activation (Fig. 8). PKNOX2 hypermethylation and silencing may serve as prognosis biomarker in primary GC.

## Materials and methods

### Human specimens and gastric cancer cell lines

Nineteen RNA samples from different human normal tissues were purchased commercially (Ambion, Austin, TX). Primary GC tissues were obtained from the Prince of Wales Hospital and Beijing Cancer Hospital and stored at −80 °C. All the samples have obtained Informed consent and the study protocol was approved by Clinical Research Ethics Committee of the Chinese University of Hong Kong and Beijing Cancer Hospital. Eighteen GC cell lines were used in this study. AGS, BGC823, KATOIII, MKN1, MKN28, MKM45, MKN74, N87, and SNU1 cell lines were obtained from ATCC (American Type Culture Collection, Manassas, VA, USA). TMK1, SNU16, SNU638, SNU719, and YCC10 cells were obtained from Korean Cell Line Bank (Seoul, Korea). HGC27, GES1, MGC803, and SGC7901 cells were purchased from Chinese Academy of Sciences (Shanghai, China). All cell lines used in this study have been authenticated and tested for mycoplasma contamination.

### Vector construction and establishment of PKNOX2 expressing and knockdown GC cells

Full-length PKNOX2 cDNA (NM_022062.2) was amplified from total RNA from human stomach (Takara Bio, USA, CA) and cloned into pcDNA3.1/myc-His A vector. The sequence of PKNOX2 cDNA insert was confirmed by sequencing. Empty vector or pcDNA3.1/myc-His A-PKNOX2 vector were transfected into AGS and MKN45 cells using Lipofectamine 2000 (Thermo Scientific), and then selected with G418 for 10–14 days. Single colonies were picked to identify cell lines stably overexpressing PKNOX2. PKNOX2 knckdown GES1 cell lines was established using shPKNOX2 plasmid. The shPKNOX2 plasmids were obtained from Ribobio Company (Guangzhou, China).

### Cell viability and colony formation assay

Cells (1 × 10^3^ cells per well) were seeded into 96 well plates. MTT assay was performed to assess cell viability. For colony formation, cells (1 × 10^3^ per well) were seeded into 6-well plates and cultured for about two weeks, followed by staining with crystal violet. Colonies with >50 cells were counted.

### Cell apoptosis and cell cycle assay

For apoptosis assay, cells were stained with Annexin V, 7-aminoactinomycin (7-AAD) double staining kit (BD Biosciences, San Jose, CA) according to the manufacturers’ protocol. For cell cycle assay, cells were fixed in cold 70% ethanol and then stained with propidium iodide (PI).

### Cell migration and invasion assays

Cell migration was evaluated by wound healing assay. In brief, cells in 6-well plates were allowed to reach confluence, and wounds were scratched using sterile tips. Wound closure was recorded every 24 h using a microscope. Cell invasion assay was performed using BioCoat™ Matrigel Invasion Chamber (Corning Falcon). Cells (4 × 10^3^) were added to the upper chamber and then placed into a 24-well plate with 0.5 mL complete medium. After incubation for 36 h, cells were stained with crystal violet and the number of invaded cells was counted under a microscope.

### In vivo tumorigenicity assay

MKN45 cells (1 × 10^6^ cells/tumor) stably expressing empty vector or PKNOX2 were injected subcutaneously into the left and right dorsal flank of 6-weeks-old male nude mice (*n* = 6), respectively. For AGS xenografts, cells stably expressing empty vector or PKNOX2 were injected subcutaneously into the left and right dorsal flank of 6-weeks-old male NOD-SCID mice (*n* = 6) (1 × 10^7^ cells/tumor), respectively. The number of animals in the two groups were determined based on our experience. Randomization was not performed as we were comparing the tumor growth of control and PKNOX2-expressing xenografts on the same mice. No blinding was performed for the animal experiments. The tumor volume was measured using a digital caliper and tumor volume was calculated as follow: volume = (shortest diameter) [2] × (longest diameter) × 0.5. At the end point, tumors were weighted and fixed in 10% formalin. All experimental procedures were approved by the Animal Ethics Committee of the Chinese University of Hong Kong.

### Methylation analyses

DNA was extracted from GC cell lines (AGS, MGC803, MKN45 and GES1) and human normal gastric tissues using the PureLink Genomic DNA Mini Kit (Thermo Fisher). Infinium Human Methylation 450 K array was performed by the Beijing Genomics Institute (Shenzhen, China). Methylation values for each CpG are expressed as β-value, which was calculated according to: methylated allele intensity/(unmethylated allele intensity + methylated allele intensity + 100). For bisulfite sequencing (BGS) analysis, bisulfite modified DNA were amplified by PCR using specific primers (Supplementary table 1). PCR products were analyzed by pyrosequencing. Percentage of methylation was calculated according to the following formula: HC/(HT + HC) × 100%, where HC is the height of the methylated peak and HT is the height of the unmethylated peak.

### PCR arrays

Human Cancer Pathway Finder PCR Array (Qiagen) and Human p53 signaling pathway PCR array were performed according to manufacturer’s instructions. Data analysis was performed using the RT [2] Profiler PCR Array Data Analysis Version 3.5 software (http://pcrdataanalysis.sabiosciences.com/).

### Chromatin-immunoprecipitation (ChIP)-PCR assay

ChIP-PCR was performed to determine the interaction between PKNOX2 and promoter regions of IGFBP5 and TP53. ChIP was performed as described [13]. Briefly, cells were cross-linked with formaldehyde for 15 min and quenched with glycine. The cells were collected, sonicated and immunoprecipitated with Myc-tag (9B11) antibody, mouse IgG or anti-PKNOX2 (ab169458) overnight at 4 °C. Samples were then washed extensively (1× low salt buffer, 1× high salt buffer, 1× LiCl buffer, and 2× TE buffer) and were eluted with 200 μL elution buffer at 37 °C for 15 min. Crosslinks were reversed by overnight incubation in 5 M NaCl at 65 °C, and were treated with RNase and Proteinase K. DNA was purified with QIA quick PCR purification kit (Qiagen). RT-PCR was used to determine the enrichment of the promoter regions of IGFBP5 and TP53. For ChIP-Seq, AGS-PKNOX2 cells were cross-linked with formaldehyde, sonicated and pulled down using anti-PKNOX2 (ab169458). Input DNA and pulled down DNA were sequenced by Novogene. Data analysis was performed by MACS2 software and Integrative Genomics Viewer browser.

### Dual-luciferase reporter assay

To evaluate p53 signaling activity, p53 luciferase reporter was co-transfected with pRL-TK (Renilla) into AGS and MKN45 cells with or without PKNOX2 expression. After 48 h, cells were lysed and luciferase activity was determined using dual-luciferase reporter assay system (Promega).

### Statistical analysis

Data were presented as mean ± S.D. Paired *t*-test was used to compare mRNA expression of PKNOX2 between tumor tissues and adjacent normal tissues. Independent samples *t*-test was utilized to analyze the difference between two groups. Receiver Operating Characteristics (ROC) curve was used to estimate the best cut-off value of the percentage PKNOX2 promoter methylation for survival analysis. Kaplan–Meier analysis and log-rank test were performed to evaluate the association between PKNOX2 methylation and overall survival. The variance was similar between the groups that were being compared. *P* *<* 0.05 was considered as statistical significance.

## Supplementary information

Supplementary Figures

Supplementary Tables

Article with changes highlighted

Conflict of interest statement
