# Supplementary material for: PKNOX2 suppresses gastric cancer through the transcriptional activation of IGFBP5 and p53
Source: Oncogene. 2019 Feb 11;38(23):4590–604. doi: 10.1038/s41388-019-0743-4 (PMC6756047; doi:10.1038/s41388-019-0743-4)
Supplement: Supplementary file 1 — Supplementary Figures [file 41388_2019_743_MOESM1_ESM.pptx]

## Slide 1
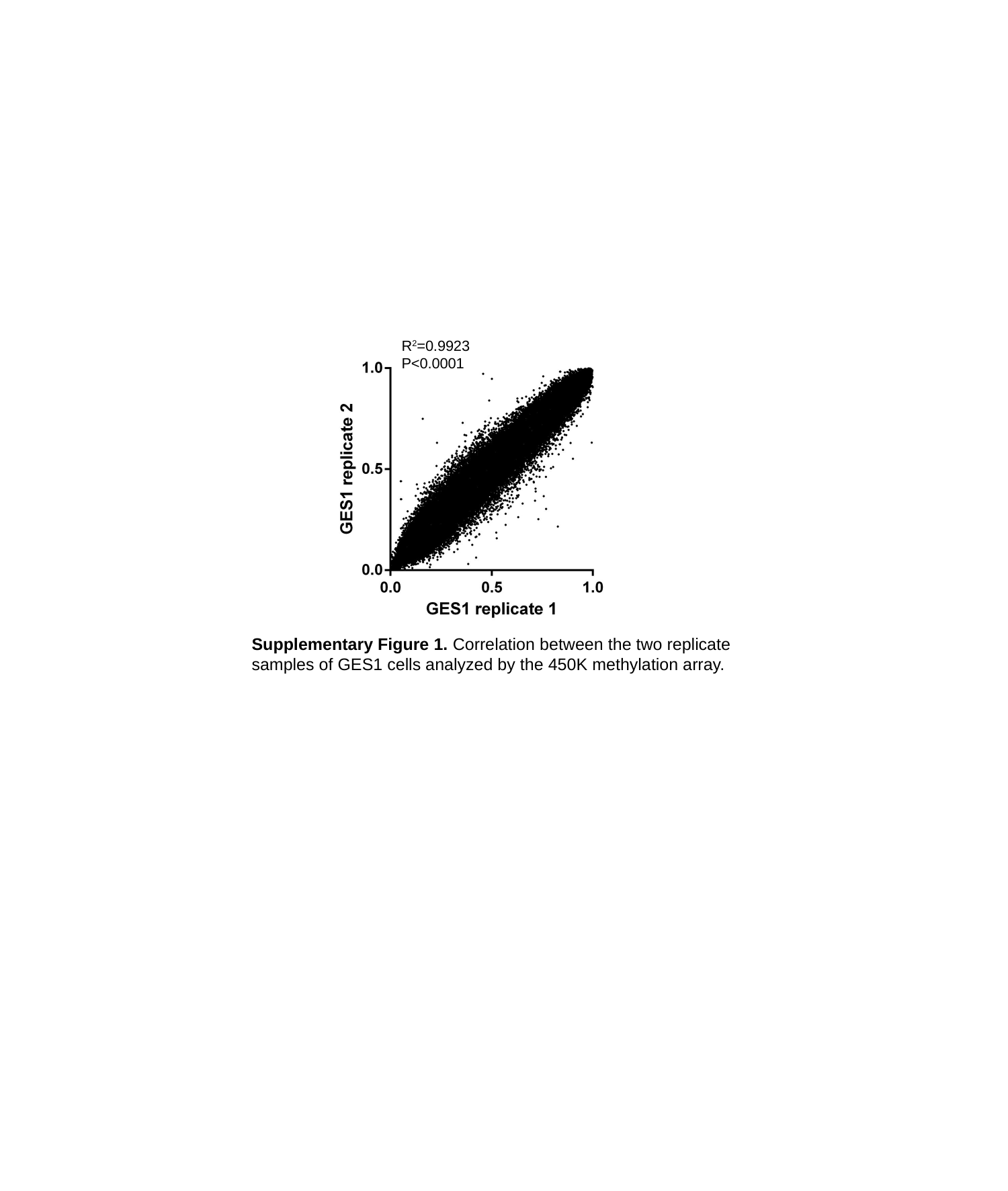

R2=0.9923
P<0.0001
Supplementary Figure 1. Correlation between the two replicate samples of GES1 cells analyzed by the 450K methylation array.

## Slide 2
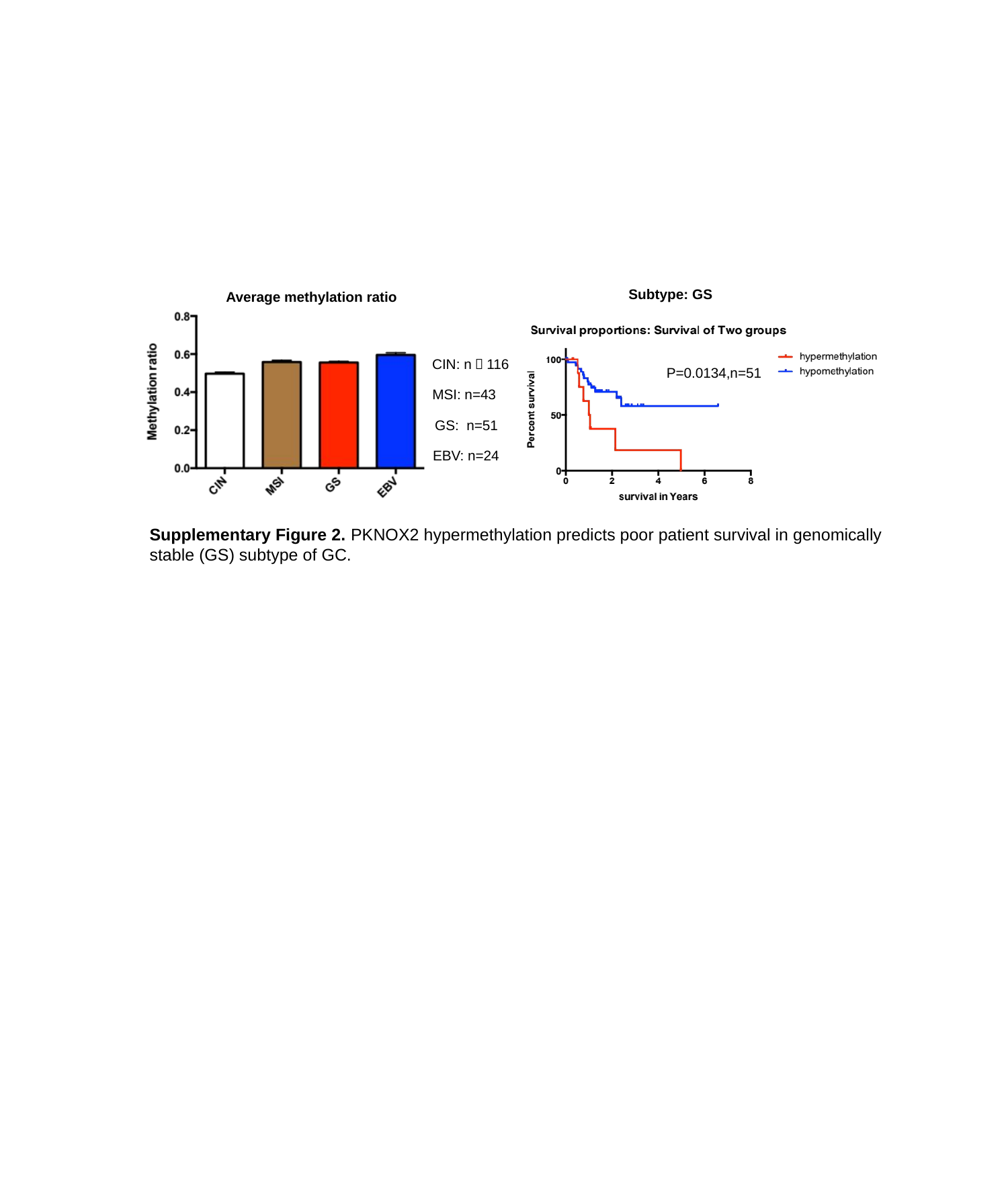

Subtype: GS
P=0.0134,n=51
Average methylation ratio
CIN: n＝116
MSI: n=43
GS: n=51
EBV: n=24
Supplementary Figure 2. PKNOX2 hypermethylation predicts poor patient survival in genomically stable (GS) subtype of GC.

## Slide 3
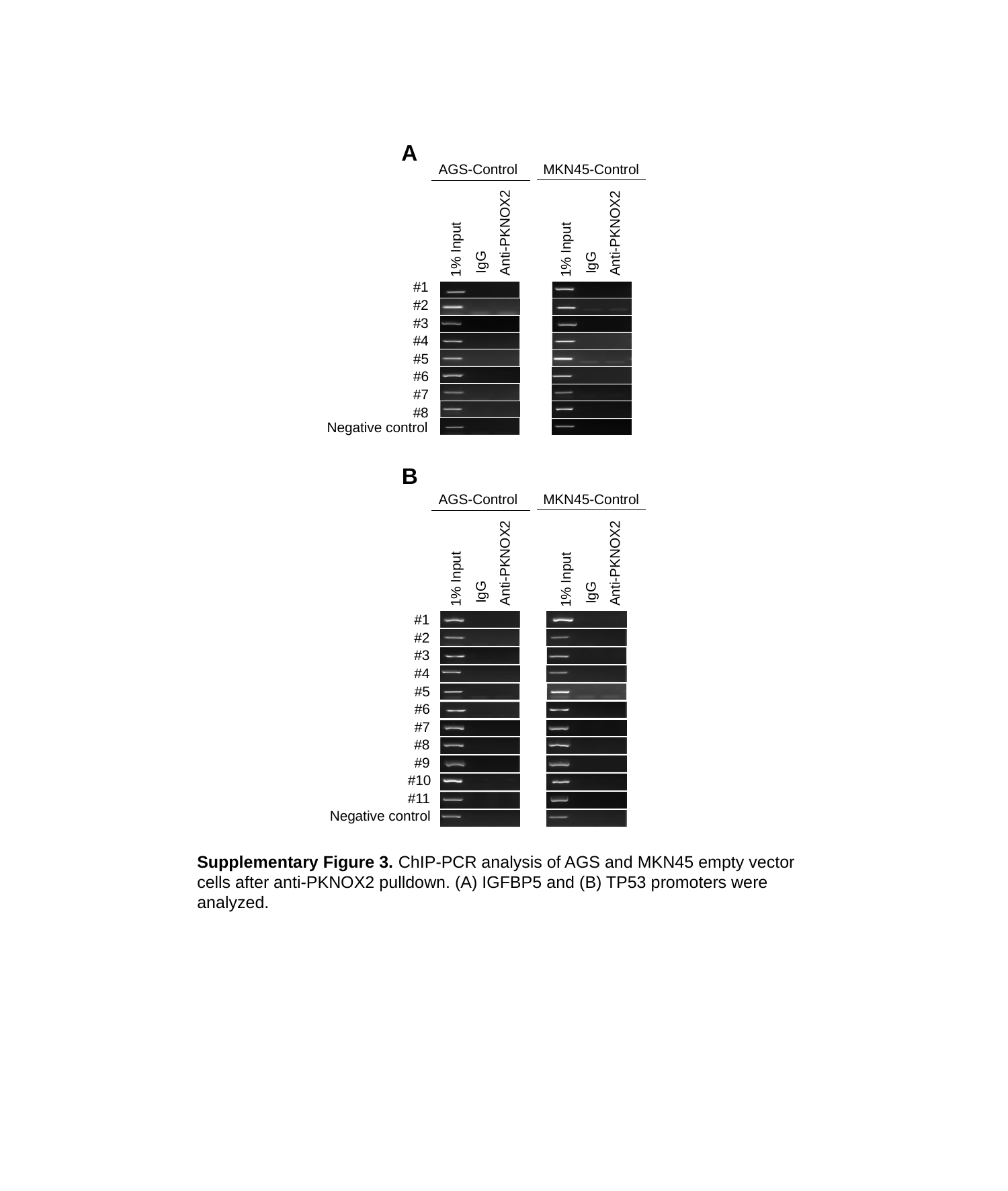

A
Anti-PKNOX2
1% Input
IgG
Anti-PKNOX2
1% Input
IgG
MKN45-Control
AGS-Control
#1
#2
#3
#4
#5
#6
#7
#8
Negative control
B
Anti-PKNOX2
1% Input
IgG
Anti-PKNOX2
1% Input
IgG
MKN45-Control
AGS-Control
#1
#2
#4
#5
#6
#7
#9
#10
#11
Negative control
#3
#8
Supplementary Figure 3. ChIP-PCR analysis of AGS and MKN45 empty vector cells after anti-PKNOX2 pulldown. (A) IGFBP5 and (B) TP53 promoters were analyzed.

## Slide 4
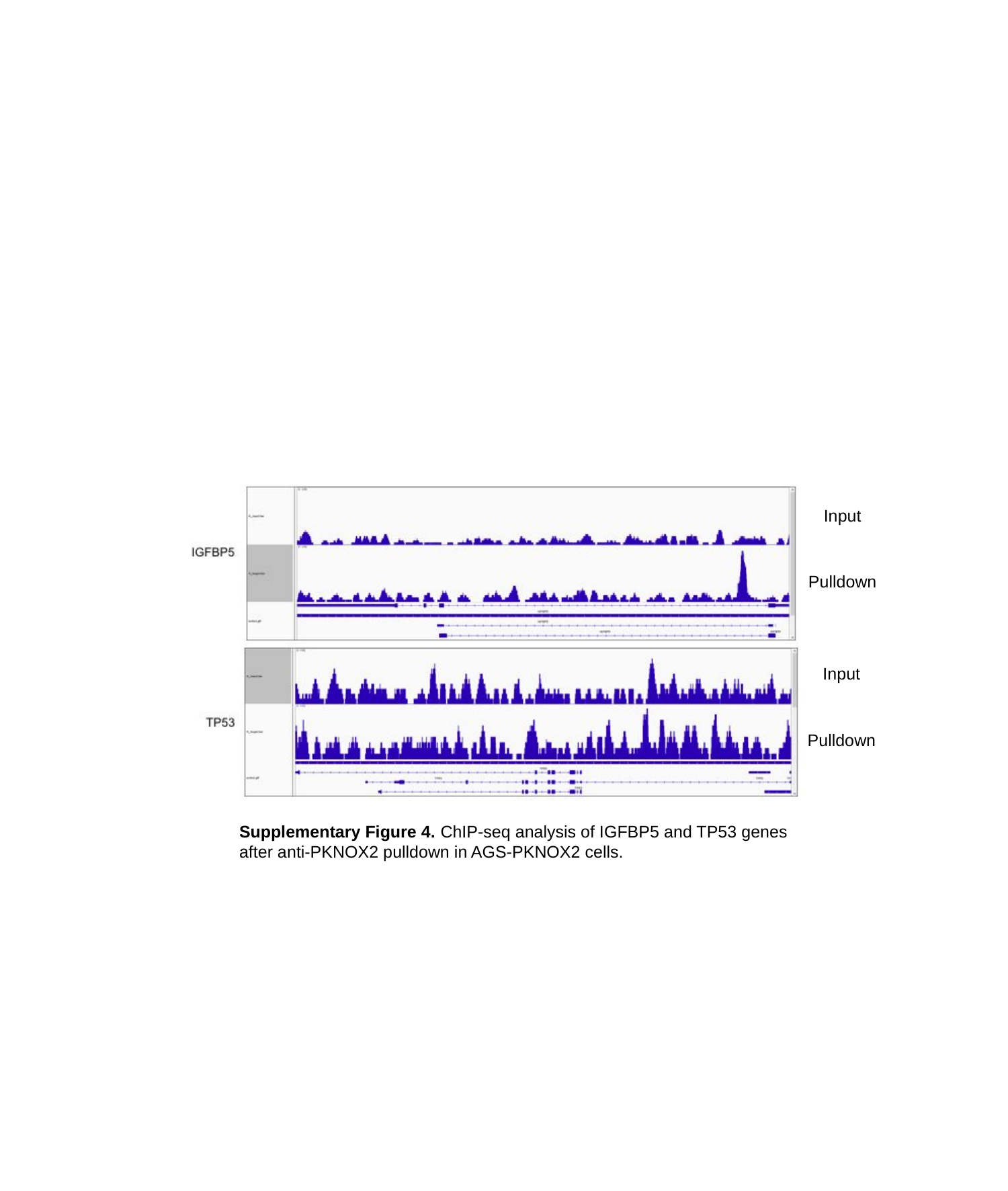

Supplementary Figure 4. ChIP-seq analysis of IGFBP5 and TP53 genes after anti-PKNOX2 pulldown in AGS-PKNOX2 cells.
Input
Pulldown
Input
Pulldown
