# Supplementary material for: PKNOX2 suppresses gastric cancer through the transcriptional activation of IGFBP5 and p53
Source: Oncogene. 2019 Feb 11;38(23):4590–604. doi: 10.1038/s41388-019-0743-4 (PMC6756047; doi:10.1038/s41388-019-0743-4)
Supplement: Supplementary file 2 — Supplementary Tables [file 41388_2019_743_MOESM2_ESM.docx]

**Supplementary Table 1.** Primers used in this study

| **Primer name** | **Sequence (5'-3')** |
| --- | --- |
| PKNOX2-F | GCAGGACTTCTGAGGCTTTCT |
| PKNOX2-R | CACATGGAGGACTGTTCGGG |
| ACTB-F | AGAGCTACGAGCTGCCTGAC |
| ACTB-R | AGCACTGTGTTGGCGTACAG |
| PKNOX2-BGS-F | ATGAGGTTAGTTAGGTAGGAAAAGG |
| PKNOX2-BGS-R | ATAAACCCCCAAAAACTCTAAAAC |
| PKNOX2-MSP-F | GGGGGTTTATCGTATTTTTTATTC |
| PKNOX2-MSP-R | ACTCCTACGACAAAAAAACG |
| PKNOX2-USP-F | TGGGGGTTTATTGTATTTTTTATTT |
| PKNOX2-USP-R | ACCTACTCCTACAACAAAAAAACAA |
| E2F4-F | TTGATCCCACACGAGAGTGC |
| E2F4-R | GGGTGGAGAAAGACGAAGCA |
| ERCC3-F | GCCATTCGACTGAACAAACCC |
| ERCC3-R | TCCGGCAGATCAAACGAAGT |
| ERCC5-F | GGTGCAGTCCGTCGTAGAA |
| ERCC5-R | CCCGGACTCCTTTAAGTGCT |
| DDB2-F | TCCTAGCAGAAGATGTGACTCA |
| DDB2-R | CACGCCAAGGATGTAGCCC |
| DDIT3-F | AGGCACTGAGCGTATCATGT |
| DDIT3-R | CTTGAACACTCTCTCCTCAGGT |
| IGFBP3-F | TCAATGTGCTGAGTCCCAGG |
| IGFBP3-R | CTTCCCCTTGGTGGTGTAGC |
| IGFBP5-F | GCCCAATTGTGACCGCAAAG |
| IGFBP5-R | GCACTGAAAGTCCCCGTCAA |
| TP53-F | GGTGGTGCCCTATGAGCC |
| TP-53R | CCAGTGTGATGATGGTGAGGA |
| BTG2-F | GCTTAAGGTCTTCAGCGGGG |
| BTG2-R | TTGATGCGAATGCAGCGGTA |
| RPRM-F | GTCCGAGGGCATGATCAACT |
| RPRM-R | TGACTCCGACAGGTTTGCTT |
| EGR1-F | GGTCAGTGGCCTAGTGAGC |
| EGR1-R | GTGCCGCTGAGTAAATGGGA |
| PIDD1-F | CTGAGCTTGGACCTGTACCC |
| PIDD1-R | GTTGCCCTCCTTTGAGGACC |
| IGFBP5-#1-F | CAGCCATTGGAAGTCGGCTA |
| IGFBP5-#1-R | GAGTGGCAGCAGAATGTGTG |
| IGFBP5-#2-F | CATTCTGCTGCCACTCTTGT |
| IGFBP5-#2-R | AGAAGTGAGGGAAATCATGTGT |
| IGFBP5-#3-F | ACACATGATTTCCCTCACTTCT |
| IGFBP5-#3-R | GCTCGTTCAGTTCAGGAGCA |
| IGFBP5-#4-F | AGTGAGATGCTCCTGAACTGAAC |
| IGFBP5-#4-R | TGTGGGCTGTTACACCTTTTAC |
| IGFBP5-#5-F | CAGGGGCAGTGACATTGGTC |
| IGFBP5-#5-R | CCTGCTTGGAAGAGGTCCTG |
| IGFBP5-#6-F | GCTCATGGCTCACTCAGGTC |
| IGFBP5-#6-R | TCCTTGTGTGTGGGGTTCTTAG |
| IGFBP5-#7-F | GGTTTTCAGACTTAGCATTTCCTCC |
| IGFBP5-#7-R | ACAACAGCATTATTGAGAGCAGTG |
| IGFBP5-#8-F | TGAAGGCAACTGGTCACCTC |
| IGFBP5-#8-R | GTTCTTCGGAGCAGGGTGAA |
| TP53-#1-F | ACCGTCAGGAGCCCTAGAAA |
| TP53-#1-R | GAGTTCAGCACCCAACCTGA |
| TP53-#2-F | TCAGGTTGGGTGCTGAACTC |
| TP53-#2-R | TGGTCCTGAGACCCACTTCT |
| TP53-#3-F | AGTTCCAAGCGCTGAAAGGA |
| TP53-#3-R | AAGTGGTGATGGCAGTGGAG |
| TP53-#4-F | CCACTTACGTGTCTCCCTCG |
| TP53-#4-R | TCCACTCCCCTGGAAACAGA |
| TP53-#5-F | TCTGTTTCCAGGGGAGTGGA |
| TP53-#5-R | CATCCTTCTCCCCACGCTTC |
| TP53-#6-F | ATTCATCGGGGAAGCGTGG |
| TP53-#6-R | AGACTCAACCGTTAGCTCCG |
| TP53-#7-F | GGATCCGACGCAGAGCTAAA |
| TP53-#7-R | AAGAATTACCGCGGGACTCG |
| TP53-#8-F | CGAGTCCCGCGGTAATTCTT |
| TP53-#8-R | AGACAGGTCTGAAGCCTGGA |
| TP53-#9-F | CAGGCTTCAGACCTGTCTCC |
| TP53-#9-R | TGCTCTCAGCTGGATCCTTT |
| TP53-#10-F | CAGCTGAGAGCAAACGCAAAAG |
| TP53-#10-R | GGGCAGAATTGGTGGAAATCAT |
| TP53-#11-F | GTATCTACGGCACCAGGTCG |
| TP53-#11-R | TTTAGCGCCAGTCTTGAGCA |

**Supplementary Table 2.** shRNA and siRNA used in this study

| **Name** | **Sequence (5'-3')** |
| --- | --- |
| shPKNOX2-1F | CAAGCAUGCCACCAAUAUA dTdT |
| shPKNOX2-1R | dTdT GUUCGUACGGUGGUUAUAU |
| shPKNOX2-2F | UGACGCUGCUGUUUGAGAA dTdT |
| shPKNOX2-2R | dTdT ACUGCGACGACAAACUCUU |
| siIGFBP5-1F: | GCCCAAUUGUGACCGCAAAtt |
| siIGFBP5-1R: | UUUGCGGUCACAAUUGGGCag |
| siIGFBP5-2F: | AGAAAGCAGUGCAAACCUUtt |
| siIGFBP5-2R: | AAGGUUUGCACUGCUUUCUct |

**Supplementary Table 3.** Antibodies used in this study

| **Antibodies** | **Source** | **Identifier** |
| --- | --- | --- |
| Anti-PKNOX2 | Abcam | ab169458 |
| GAPDH | Santa Cruz | sc-25778 |
| PARP Antibody | Cell Signaling Technology | #9542 |
| Cleaved PARP | Cell Signaling Technology | #9541 |
| p27 Kip1 | Cell Signaling Technology | #3686 |
| p53 | Santa Cruz | sc-126 |
| β-actin | Santa Cruz | sc-47778 |
| Caspase-3 | Cell Signaling Technology | #9665 |
| Caspase-7 | Cell Signaling Technology | #9492 |
| Caspase-8 | Cell Signaling Technology | #9746 |
| Caspase-9 | Cell Signaling Technology | #9508 |
| CDK4 | Cell Signaling Technology | #12790 |
| cleaved caspase 8 | Cell Signaling Technology | #9496s |
| cleaved caspase 9 | Cell Signaling Technology | #9501s |
| Cleaved Caspase-3 | Cell Signaling Technology | #9661 |
| Cleaved Caspase-7 | Cell Signaling Technology | #9491 |
| Cleaved PARP | Cell Signaling Technology | #5625 |
| c-Myc | Santa Cruz | #sc-40 |
| cyclin D1 | Cell Signaling Technology | #2922S |
| Lamin A/C | Cell Signaling Technology | #4777 |
| N-Cadherin | Cell Signaling Technology | #4061S |
| p21 (F-5) | Santa Cruz | sc-6246 |
| PARP | Cell Signaling Technology | #9532 |
| Myc-tag | Cell Signaling Technology | #2276（9B11） |
| E-cadherin | Cell Signaling Technology | #14472 |
| Vimentin | Cell Signaling Technology | #5741 |
| Slug | Cell Signaling Technology | #9585 |
| IGFBP5 | Abcam | ab4257 |
