# Supplementary material for: PKNOX2 suppresses gastric cancer through the transcriptional activation of IGFBP5 and p53
Source: Oncogene. 2019 Feb 11;38(23):4590–604. doi: 10.1038/s41388-019-0743-4 (PMC6756047; doi:10.1038/s41388-019-0743-4)
Supplement: Supplementary file 4 — Conflict of interest statement [file 41388_2019_743_MOESM4_ESM.docx]

The authors declared no conflict of interest.
